# UBE2T promotes glioblastoma invasion and migration via stabilizing GRP78 and regulating EMT

**DOI:** 10.18632/aging.103239

**Published:** 2020-06-03

**Authors:** Peng Huang, Yuduo Guo, Zitong Zhao, Weihai Ning, Haoran Wang, Chunyu Gu, Mingshan Zhang, Yanming Qu, Hongwei Zhang, Yongmei Song

**Affiliations:** 1 Department of Neurosurgery, Sanbo Brain Hospital, Capital Medical University, Beijing 100021, China; 2 State Key Laboratory of Molecular Oncology, National Cancer Center, National Clinical Research Center for Cancer, Cancer Hospital, Chinese Academy of Medical Sciences and Peking Union Medical College, Beijing 100021, China

**Keywords:** glioblastoma, therapeutic target, epithelial-mesenchymal transition, Ubiquitin-conjugating enzyme E2T, Endoplasmic Reticulum Lumenal Ca(2+)-Binding Protein Grp78

## Abstract

Glioblastoma (GBM) generally has a dismal prognosis, and it is associated with a poor quality of life as the disease progresses. However, the development of effective therapies for GBM has been deficient. Ubiquitin-conjugating enzyme E2T (UBE2T) is a member of the E2 family in the ubiquitin-proteasome pathway and a vital regulator of tumour progression, but its role in GBM is unclear. In this study, we aimed to clarify the role of UBE2T in GBM. Bioinformatics analysis identified UBE2T as an independent risk factor for gliomas. Immunohistochemistry was used to measure UBE2T expression in GBM and normal tissue samples obtained from patients with GBM. The effects of UBE2T on GBM cell invasion and migration were analysed using the Transwell assay. BALB/c nude mice were used for the in vivo assays. Immunoblotting and immunoprecipitation were performed to determine the molecular mechanisms. UBE2T was highly expressed in GBM tissues, and its expression was linked to a poor prognosis. In vitro, depletion of UBE2T significantly suppressed cell invasion and migration. Moreover, UBE2T depletion suppressed the growth of GBM subcutaneous tumours in vivo. Further experiments revealed that UBE2T suppressed invasion and migration by regulating epithelial- mesenchymal transition (EMT) via stabilising GRP78 in GBM cells. We uncovered a novel UBE2T/GRP78/EMT regulatory axis that modulates the malignant progression and recurrence of GBM, indicating that the axis might be a valuable therapeutic target.

## INTRODUCTION

Malignant gliomas are the most common fatal brain tumours. Glioblastoma (GBM), the most aggressive glioma, is associated with a dismal prognosis and poor quality of life, as well as an average survival of 10–14 months after diagnosis over the last 5 years [1, 2]. GBM is highly invasive, infiltrating the surrounding brain parenchyma, but the lesions are typically confined to the central nervous system [3, 4]. The survival of patients with GBM remains poor despite standard surgery, radiation and chemotherapy [5]. Recently, large-scale genome-wide profiling studies yielded an abundance of genome data and provided deeper insights into the molecular pathogenesis of glioma [2, 6]. This increased understanding of the genetic and pathogenic mechanisms of GBM has stimulated the development of novel therapeutic strategies, including the use of molecular targeted agents [7, 8]. For example, the DNA-alkylating agent temozolomide (TMZ) in combination with radiotherapy has displayed a good therapeutic effect in patients with tumours displaying promoter methylation of the DNA repair enzyme O6-methylguanine-DNA methyl transferase [2, 9, 10].

Genetic and epigenetic alterations are related to the genesis and progression of various types of tumours [11, 12]. Post-translational modification, through which the ubiquitin proteasome system regulates protein ubiquitination and stability, is recognised as a key regulator of cell proliferation, invasion, differentiation and death [13, 14]. Ubiquitin-conjugating enzyme E2T (UBE2T) is a member of the E2 family in the ubiquitin-proteasome pathway. Ubiquitylation plays role in protein interactions, localization and enzymatic activities, further affecting cellular processes, including transcription, DNA damage signalling and DNA repair, cell cycle progression, endocytosis, apoptosis and various other processes [15]. UBE2T plays a crucial role in cell cycle progression, signal transduction and tumorigenesis [16, 17]. UBE2T over-expression has been found in different tumour types. In particular, UBE2T over-expression may contribute to breast carcinogenesis throughout the down-regulation of BRCA1 [18]. UBE2T is over-expressed in bladder cancers, and UBE2T depletion significantly suppresses the proliferation and colony formation of bladder cancer cells [19]. UBE2T is up-regulated in HCC, and it exerts oncogenic exerts via p53 ubiquitination [20]. However, the role and clinical significance of UBE2T in GBM remain unclear.

To further confirm the intersection between tumorigenesis and recurrence in GBM, we found that UBE2T is simultaneously over-expressed in primary and recurrent GBM. Mechanistically, UBE2T promotes the invasion and migration of GBM through stabilising GRP78 and regulating epithelial-mesenchymal transition (EMT) biomarkers. These results identify GRP78 as a substrate of UBE2T and extend our understanding of the tumour mechanism-related functions of UBE2T in GBM.

## RESULTS

### UBE2T is an independent risk factor for gliomas

**Figure 1:**
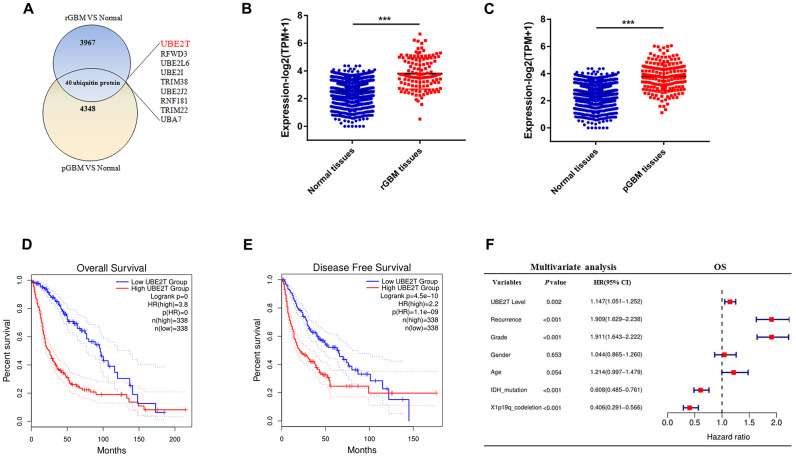
**Ubiquitin-conjugating enzyme E2T (UBE2T) is an independent risk factor for gliomas.** (**A**) Overlapping differentially expressed genes of recurrent and primary glioblastoma (GBM) compared with the findings in normal brain tissues. (**B**) UBE2T expression in recurrent GBM and normal tissues from the Genotype-Tissue Expression Project (GTEx) and Chinese Glioma Genome Atlas (CGGA) databases. (**C**) UBE2T expression in primary GBM and normal tissues from the GTEx and CGGA databases. (**D**) Overall survival analysis according to UBE2T expression in glioma using the GEPIA tool. (**E**) Disease-free survival analysis according to UBE2T expression in glioma using the GEPIA tool. (**F**) Multivariate analysis of the relationship of UBE2T with overall survival in glioma using the CGGA RNA sequencing dataset.

Post-translational modification with ubiquitin controls many processes for development, cell division and migration in cancer [21]. We sought to identify ubiquitylation enzymes that were highly expressed in GBM. For this purpose, we formulated some standards to screen candidate genes. (I): First, we examined the RNA sequencing data of glioma and normal brain tissues from the CGGA and GTEx databases. After data consolidation, batch normalisation and normalisation of the expression profiles, 4007 DEGs involved in recurrent GBM and 4388 DEGs involved in primary GBM were identified using the edgrR package (adjusted *p* < 0.001, |logFC| > 1). (II) A total of 3829 DEGs for both recurrent and primary GBM were selected for further analysis (Supplementary Table 1A). From these DEGs, 40 ubiquitylation enzymes were selected for further study (Figure 1A). (III) We then aimed to identify ubiquitylation enzymes that have not been reported but have clinical significance in glioma. We identified nine ubiquitylation enzymes (Figure 1A), namely UBE2L6, UBE2I, RFWD3, TRIM38, UBE2J2, UBE2T, RNF181, TRIM22 and UBA7. Compared with the findings in normal tissues, UBE2T expression was higher in both primary and recurrent GBM in the CGGA and GTEx databases (Figure 1B, 1C). To evaluate the prognostic value of the candidate genes in patients, log-rank survival curve analysis was performed using the GEPIA tool. The results illustrated that the overall and disease-free survival were shorter in patients with high UBE2T expression than in those with low UBE2T expression (Figure 1D, 1E). Then, multivariate Cox regression analysis was performed to further estimate the prognostic value of the candidate genes. The analysis revealed that UBE2T expression is an independent prognostic biomarker for patients with glioma (Figure 1F). On the basis of these findings, we focused on UBE2T. The results suggest that UBE2T may play an oncogenic role in GBM.

### UBE2T expression is higher in GBM and it promotes cell invasion and migration

**Figure 2:**
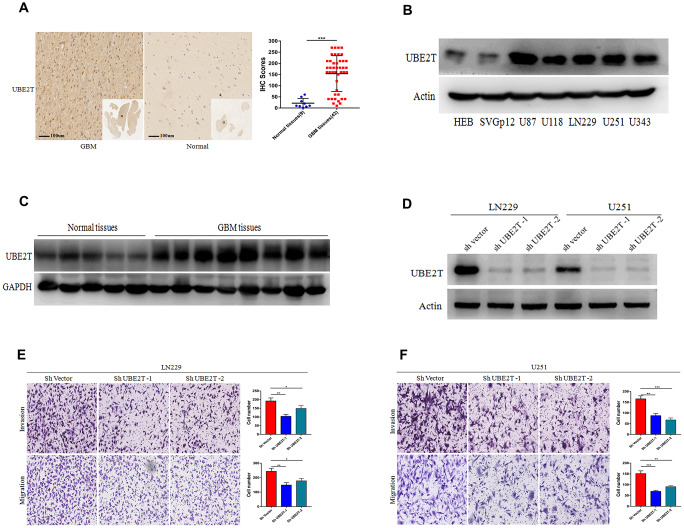
**Ubiquitin-conjugating enzyme E2T (UBE2T) expression is higher in glioblastoma (GBM) and it promotes cell invasion and migration.** (**A**) Immunohistochemical staining of UBE2T in human GBM tissues compared with the findings in normal brain tissues (×100). Scale bar, 100 μm. (**B**) Immunoblotting of UBE2T protein levels in normal human astrocytes (HEB and SVG p12) and GBM cell lines (U87, U118, LN229, U251 and U343). Expression levels were normalized to those of β-actin. (**C**) Immunoblotting of UBE2T protein levels in normal brain and GBM tissues. Expression levels were normalized to those of GAPDH. (**D**) Transfection efficacy of UBE2T short hairpin RNA (shRNA) in LN229 and U251 cells was analysed via immunoblotting. (**E**, **F**) The effects of UBE2T shRNA on the invasion and migration of LN229 (**E**) and U251 (**F**) cells. (Error bars indicate the SEM of three independent experiments. Two-tailed Student’s t-test. *, P < 0.05; **, P < 0.01; ***, P < 0.001).

To further explore the protein expression of UBE2T in GBM, we assessed its expression in normal and GBM tissues via IHC. Compared with the findings in normal tissues (median IHC score = 10), UBE2T was over-expressed in GBM tissues (median IHC score = 180) (Figure 2A). Immunoblotting revealed that GBM cells exhibit higher UBE2T protein levels than normal cells (Figure 2B). Furthermore, immunoblotting demonstrated that UBE2T expression was higher in frozen GBM tissue than in frozen normal brain tissue (Figure 2C). To explore the biological function of UBE2T in GBM progression, LN229 and U251 cells with stable UBE2T depletion were established. UBE2T expression was effectively down-regulated in shRNA-transfected cells (Figure 2D), leading to significant decreases of invasion and migration in both shUBE2T-1– and shUBE2T-2–transfected LN229 and U251 compared with the findings in cells transfected with ShVector (Figure 2E, 2F).

### UBE2T interacts with GRP78 and GRP78 is an independent risk factor for gliomas

**Figure 3:**
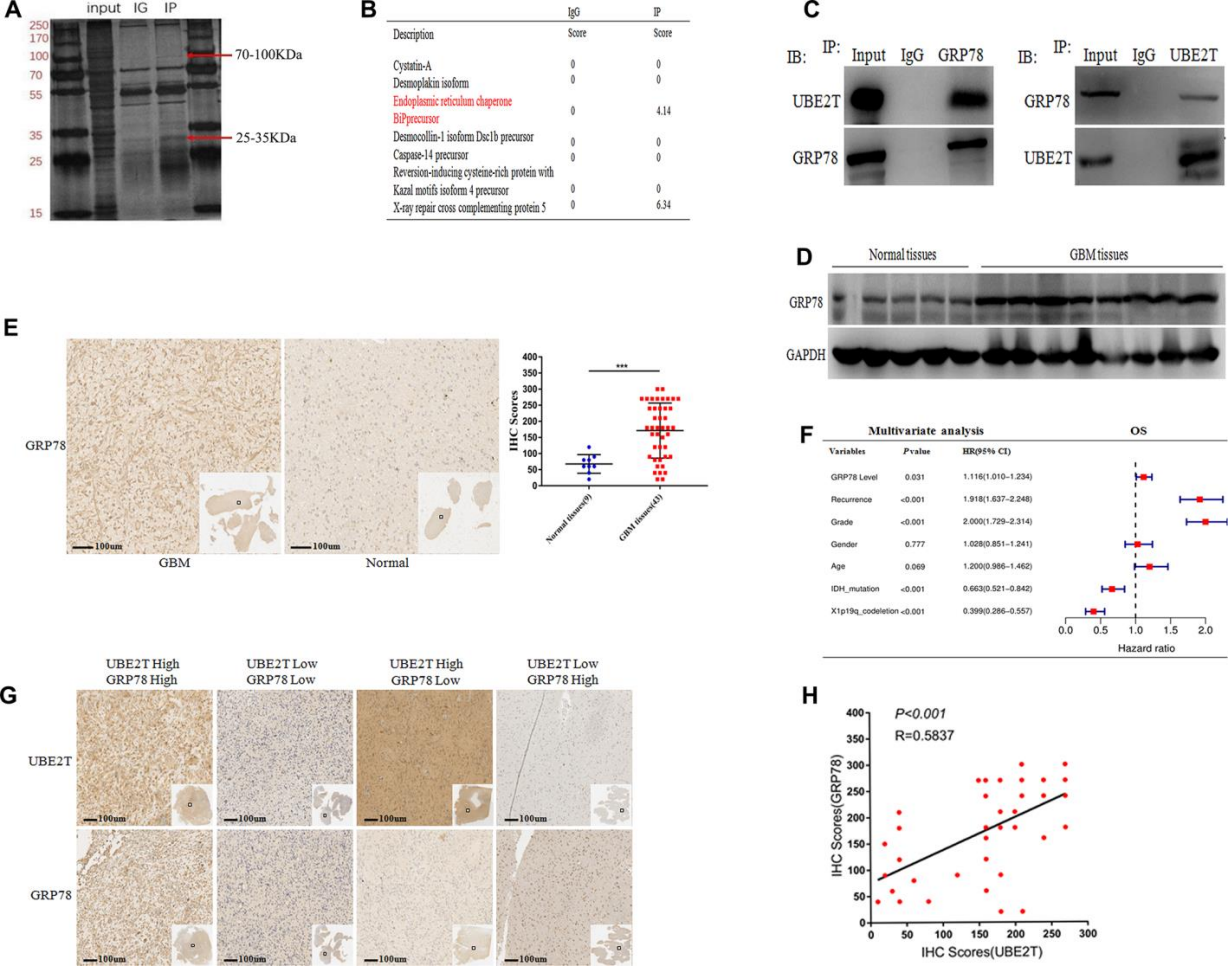
**Ubiquitin-conjugating enzyme E2T (UBE2T) interacts with GRP78 and GRP78 is an independent risk factor for gliomas.** (**A**) SDS-PAGE analysis of UBE2T immunoprecipitates. The indicated band was excised and examined via mass spectrometry. (**B**) The mass spectrometry result allowed the identification of proteins. (**C**) U251 cell lysates were incubated with Protein A/G Sepharose conjugated with anti-UBE2T and anti-GRP78 antibodies. The immunoprecipitates were detected via immunoblotting. (**D**) Immunoblotting of GRP78 protein levels in normal brain and GBM tissues. Expression levels were normalized to those of GAPDH. (**E**) Immunohistochemistry of GRP78 in human GBM tissues compared with that in normal brain tissues (×100). Scale bar, 100 μm. (**F**) Multivariate analysis of the relationship of GRP78 with overall survival (OS) in patients with glioma using the Chinese Glioma Genome Atlas RNA sequencing dataset. (**G**) Representative images of the immunohistochemical staining of UBE2T and GRP78 in glioblastoma (n = 43, ×100). Scale bar, 100 μm. (**H**) The correlation between UBE2T and GRP78 protein levels was analysed in glioblastoma tissues. The samples were classified into low and high expression groups based on the UBE2T and GRP78 IHC scores. The protein expression level of GRP78 was positively correlated with the GRP78 expression level. Each dot indicates the relative protein expression level. Data were analysed using Pearson’s correlation coefficient. (Error bars indicate the SEM of three independent experiments. Two-tailed Student’s *t*-test. *, P < 0.05; **, P < 0.01; ***, P < 0.001).

We next aimed to reveal the molecular mechanisms by which UBE2T promotes the tumorigenicity of GBM. We discovered the interacting partners of UBE2T via mass spectrometry (Figure 3A). As shown in Figure 3B, the potential interacting proteins were GRP78 and XXCR5, which play crucial roles in tumorigenesis, making them worthy of further examination [22]. Co-IP assays were conducted to confirm that UBE2T binds to its putative protein partners. Regarding the two proteins, GRP78 was the only protein that bound to UBE2T (Figure 3C). Moreover, we further found that GRP78 expression was higher in frozen GBM tissues than in frozen normal brain tissues via immunoblotting (Figure 3D). Through IHC assays, we assessed GRP78 expression in normal and GBM tissues. Compared with the findings in normal tissues (median IHC score = 60), GRP78 was over-expressed in GBM tissues (median IHC score = 160) (Figure 3E). Multivariate Cox regression analysis uncovered that GRP78 expression is an independent prognostic biomarker for patients with gliomas (Figure 3F). Additionally, the IHC assay of UBE2T and GRP78 expression in GBM tissues were performed and statistical analysis about the relationship between the expression level (Figure 3G, 3H). Interestingly, a positive correlation was observed between UBE2T expression and GRP78, the spearman r^2^ being 0.58 with *P* < 0.001. Collectively, these results demonstrated that the tumorigenicity function of UBE2T in GBM could require binding with GRP78.

### UBE2T enhances GBM invasion and migration via GRP78

**Figure 4:**
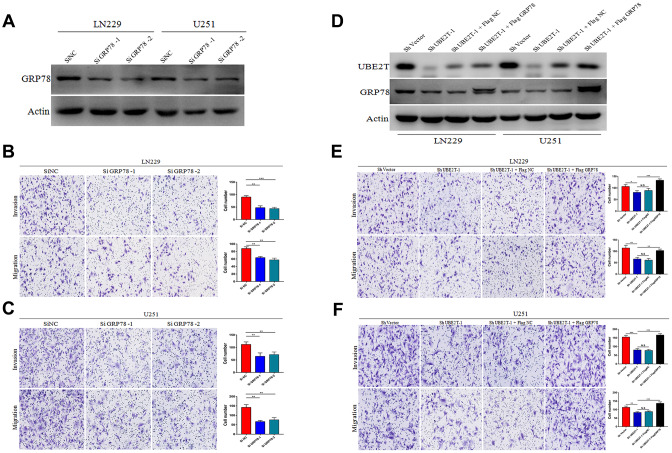
**Ubiquitin-conjugating enzyme E2T (UBE2T) enhances glioblastoma (GBM) invasion and migration via GRP78.** (**A**) Transfection efficacy of GRP78 siRNA in LN229 and U251 cell lines was analysed via immunoblotting. (**B**, **C**) The effects of GRP78 siRNA on the invasion and migration of LN229 (B) and U251 (C) cells. (**D**) UBE2T was depleted in GRP78–over-expressing LN229 and U251 cells. Cell lysates were analysed via immunoblotting using the indicated antibodies. (**E**, **F**) The effects of UBE2T depletion on the invasion and migration of GRP78–over-expressing LN229 (**E**) and U251 (**F**) cells. (Error bars indicate the SEM of three independent experiments. Two-tailed Student’s *t*-test. *, P < 0.05; **, P < 0.01; ***, P < 0.001).

We then examined whether UBE2T contributes to GBM malignant transformation and progression by regulating GRP78. First, we transfected LN229 and U251 cells, which express low levels of GRP78, with GRP78 or control siRNA. The transfection efficiency in each cell line was examined via immunoblotting (Figure 4A). Cell invasion and migration were measured 48 h after transfection using Transwell assays. We found that depletion of GRP78 markedly decreased migration and invasion in LN229 (Figure 4B) and U251 cells (Figure 4C) compared with the control findings. Second, we over-expressed GRP78 via transfection of FLAG-GRP78 over-expression plasmids into UBE2T-depleted LN229 and U251 cells. The transfection efficiency in each cell line was examined via immunoblotting (Figure 4D), and we observed that the FLAG-GRP78 over-expression enhanced the invasion and migration of UBE2T-depleted LN229 (Figure 4E) and U251 cells (Figure 4F). These results illustrated that GRP78 over-expression rescued the effects of UBE2T depletion and strengthened tumour development.

### UBE2T maintains the stability of GRP78 and regulates EMT markers

**Figure 5:**
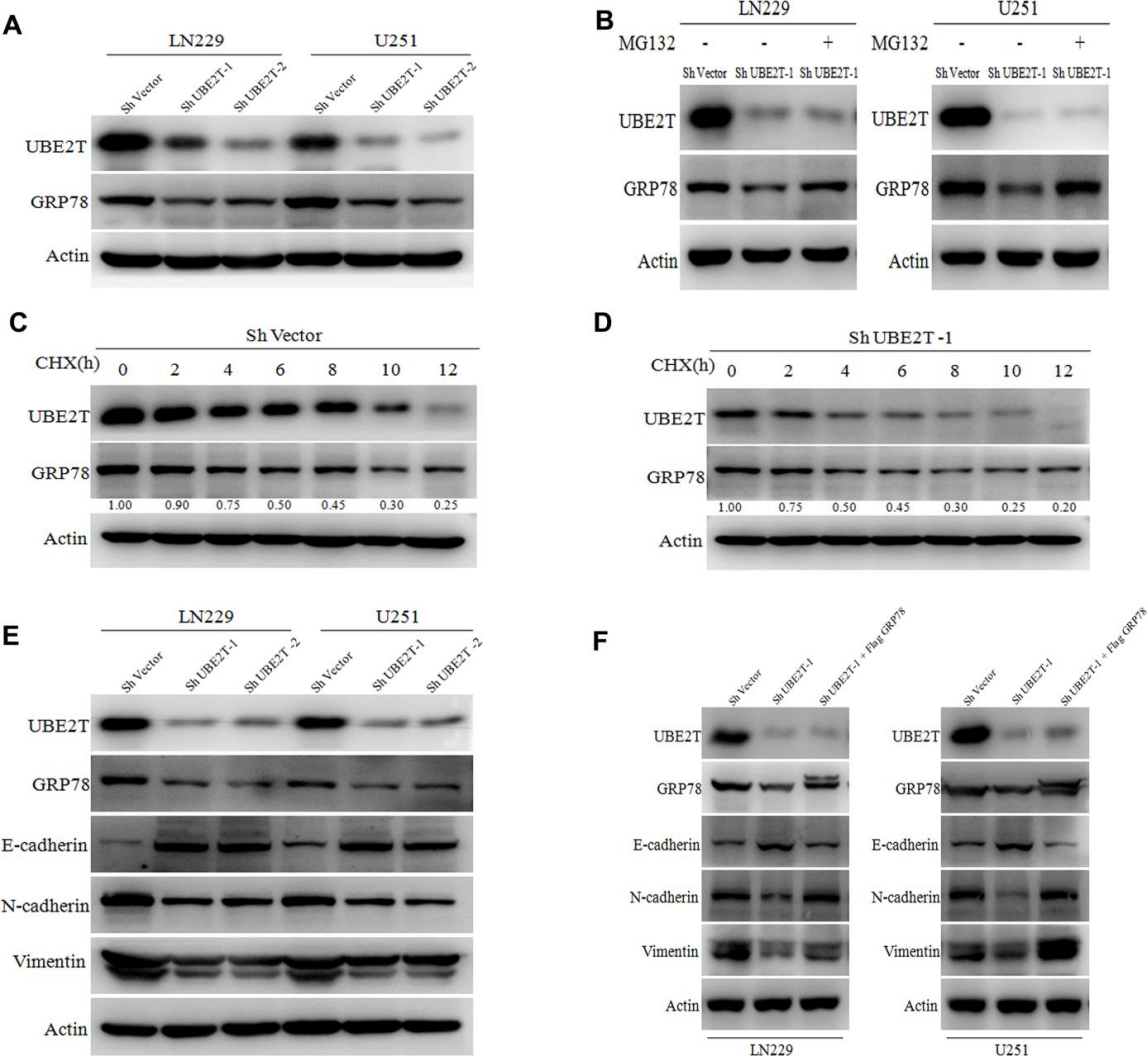
**Ubiquitin-conjugating enzyme E2T (UBE2T) maintains GRP78 stability and regulates epithelial-mesenchymal transition (EMT) markers.** (**A**) UBE2T was depleted in LN229 and U251 cells. Cell lysates were examined using the indicated antibodies. (**B**) Immunoblotting of UBE2T, GRP78 and actin in LN229 and U251 cells transduced with UBE2T short hairpin RNA (shRNA) in the absence or presence of 10 μmol/L MG132 for 8 h. (**C**) U251 cells transfected with the control shRNA vector (ShVector) were treated with cycloheximide (100 μg/mL) and collected at the indicated times for immunoblotting. Quantification of GRP78 expression relative to β-actin expression is shown. (**D**) U251 cells transfected with ShUBE2T-1 were treated with cycloheximide (100 μg/mL) and collected at the indicated times for immunoblotting. Quantification of GRP78 expression relative to β-actin expression is shown. (**E**) Immunoblotting of UBE2T, GRP78, actin and EMT biomarkers (E-cadherin, N-cadherin and vimentin) in LN229 and U251 cells transfected with ShUBE2T-1, ShUBE2T-2 or ShVector. (**F**) Immunoblotting of UBE2T, GRP78, N-cadherin, E-cadherin, vimentin and Actin in LN229 and U251 cells transduced with the indicated plasmids. (Error bars indicate the SEM of three independent experiments).

Regarding the possible regulatory effects of UBE2T on GRP78, we found that UBE2T depletion remarkably decreased GRP78 protein expression (Figure 5A). This effect was restored by treatment with the proteasome inhibitor MG132 in LN229 and U251 cells (Figure 5B). To further validate that UBE2T affects GRP78 protein stability, we treated the indicated cells with the protein synthesis inhibitor cycloheximide (CHX), and cells were collected after 0, 2, 4, 6, 8, 10 and 12 h of exposure. Notably, compared with the findings in cells transfected with the control vector, UBE2T depletion led to a shortened half-life of GRP78 protein (ShVector, 6 h; ShUBE2T-1, 4 h) (Figure 5C, 5D). To further explore the mechanisms by which UBE2T increase cell invasion and migration, we transfected LN229 and U251 cells with shRNA targeting UBE2T. Depletion of UBE2T resulted in significantly increased levels of the epithelial biomarker E-cadherin and simultaneously decreased levels of the mesenchymal biomarkers N-cadherin and vimentin (Figure 5E). Furthermore, GRP78 over-expression in these cell lines reversed the effects of shRNA transfection on EMT biomarker expression (Figure 5F). These findings further support the possibility that GRP78 is a downstream and functional target of UBE2T.

### The function of UBE2T was uncovered in vivo

**Figure 6:**
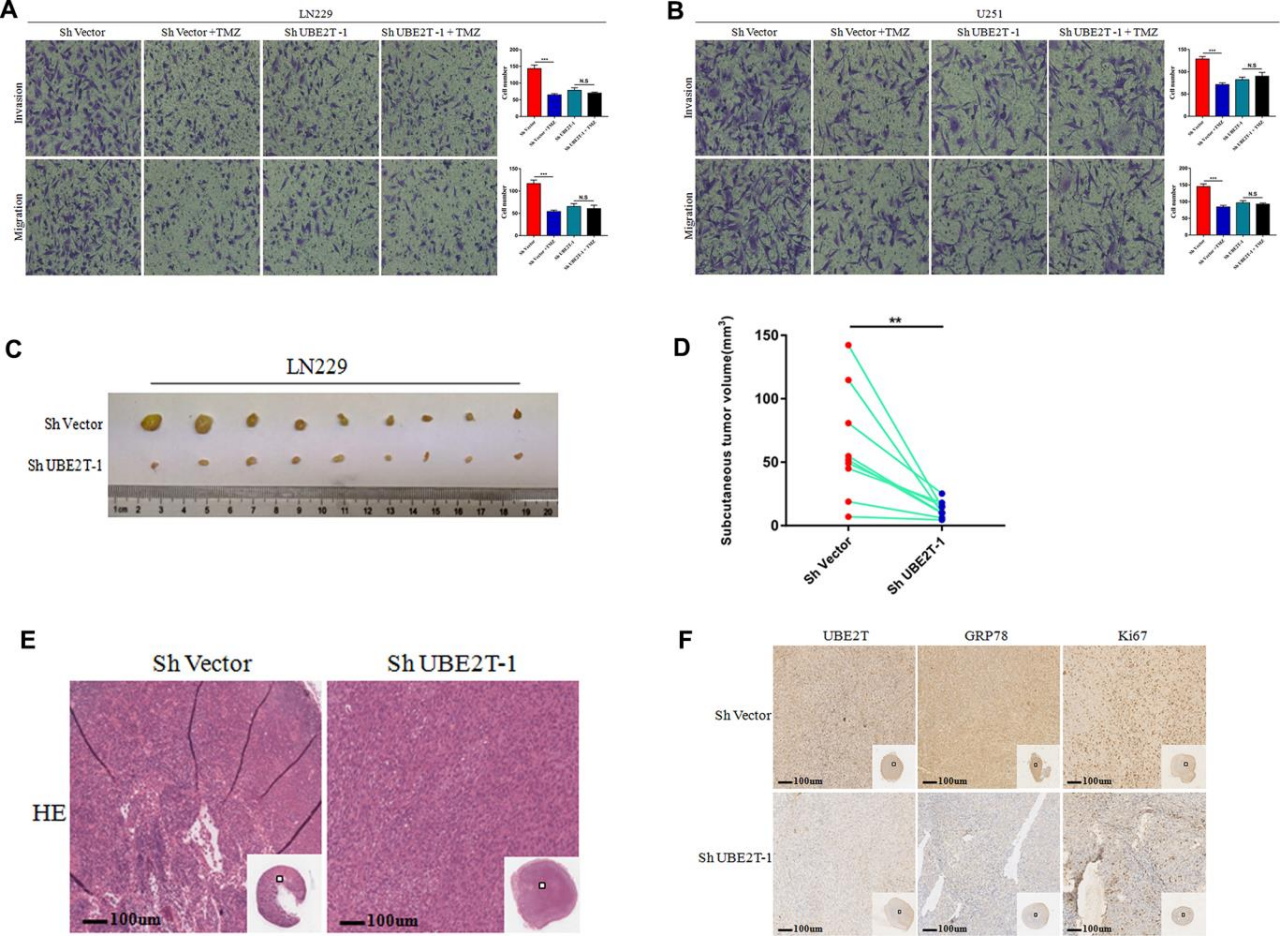
**The function of Ubiquitin-conjugating enzyme E2T (UBE2T) was uncovered in vivo.** (**A**, **B**) The effects of transfection with short hairpin RNA (shRNA) targeting UBE2T-1, treatment with temozolomide (TMZ, 200 μM) or both on the invasion and migration of LN229 (**A**) and U251 (**B**) cells. (**C**, **D**) LN229 cells transfected with ShUBE2T-1 or the control vector (Sh Vector) were subcutaneously injected (5 × 10^6^ cells per mouse) into 6-week-old nude mice (n = 9). Representative images (left) (**C**) and quantitative analysis (right) (**D**) of subcutaneous tumour volumes. (**E**) Haematoxylin and eosin (HE) staining was performed to illustrate the tumour cytostructure. (**F**) Immunohistochemical staining of UBE2T, GRP78 and Ki-67 in ShUBE2T-1 and ShVector subcutaneous tumour samples (×100). Scale bar, 100 μm. (Error bars indicate the SEM of three independent experiments. Two-tailed Student’s *t*-test. *, P < 0.05; **, P < 0.01; ***, P < 0.001).

The aforementioned data indicate that UBE2T maintains GRP78 stability and enhances GBM cell invasion and migration by regulating EMT biomarkers. Further tests were conducted to assess the effects of UBE2T depletion in combination with chemotherapy. In this aim, LN229 and U251 cells transfected with ShVector or ShUBE2T-1 cells were incubated with 200 μM TMZ. TMZ effectively attenuated the invasive and migratory potential of LN229 (Figure 6A) and U251 cells (Figure 6B). However, TMZ had no effect on the malignant phenotypes of UBE2T-depleted LN229 (Figure 6A) and U251 cells (Figure 6B). Then, LN229 cells transfected with ShVector or ShUBE2T-1 were transplanted into nine pairs of nude mice. Imaging (Figure 6C), and quantitative analysis (Figure 6D) revealed significant differences in the tumour volume between the two groups of mice. The change curve of tumor volume showed in Supplementary Figure 1. Images of haematoxylin and eosin-stained subcutaneous tumours are presented in Figure 6E. Brisk mitotic activity was weakened in ShUBE2T-1–transplanted mice. Depletion of UBE2T potently reduced the levels of Ki-67 and GRP78 in subcutaneous tumours (Figure 6F). Notably, UBE2T functions as a tumour promoter in GBM in vivo, making it a potential therapeutic target.

## DISCUSSION

Despite the use of combination treatment with surgery, chemotherapy and radiotherapy for decades, the prognosis for patients with GBM remains poor. Thus, there is a substantial need for novel therapeutic targets [23]. We analysed RNA sequencing data from the CGGA and GTEx datasets [24, 25]. It was determined that UBE2T expression was significantly up-regulated in primary and recurrent GBM samples compared with that in normal samples. UBE2T is an independent risk factor for gliomas. Previous studies demonstrated that UBE2T over-expression promotes cancer development. However, whether UBE2T could promote the occurrence and development of GBM was unclear.

In this study, we found that GBM tissues had higher UBE2T protein levels than normal tissues. In vitro and in vivo assays demonstrated the effects of UBE2T on GBM cell invasion and migration, and the protein also promoted GBM proliferation in a mouse model. We identified a novel UBE2T downstream substrate, namely GRP78, a regulator that could promote tumorigenesis, metastasis and drug resistance in cancers [26, 27]. GRP78 is a resident chaperone of the endoplasmic reticulum and a master regulator of the unfolded protein response under physiological and pathological cell stress conditions. However, the relation between these two proteins has not been studied. UBE2T belongs to the ubiquitine proteasome system. The sequential enzymatic processes that covalently attach ubiquitin, a 76 residue polypeptide, to target proteins and chains involving internal ubiquitin lysine residues K6, K11, K27, K29, K33, K48, K63 [28]. Different modifying ubiquitin lysine residues play different functions. It is well known that polyubiquitin chains, in particular those linked through K48 and K63, play a key role in the regulation of the tumor progression [29]. For example, Lys (K) 63-linked ubiquitination modulated by Ube2v1 expression enhanced protein aggregation and contributed to Ube2v1's function in regulating protein aggregate formation [30]. However, TRIM3 could directly interact with Beclin1, and improved its K48-linked polyubiquitinaion, leading to the degradation of Beclin1 and then regulated autophagy [31]. We further demonstrated that UBE2T functions as a tumour promoter in GBM by maintaining the protein level of GRP78. Further testing revealed that GRP78 is frequently over-expressed in GBM and that UBE2T maintained the stability of GRP78, leading to GBM cell invasion and migration in vitro. UBE2T might stable GRP78 via Lys (k) 63-linked ubiquitination. However, the combination of TMZ treatment and UBE2T depletion had no effect on these malignant phenotypes. In summary, we propose a model in which UBE2T promotes the development of GBM through the UBE2T/GRP78 axis, suggesting its potential as a novel treatment target.

Surprisingly, we found that the protein expression of EMT markers is regulated by the UBE2T/GRP78 axis in GBM cells. UBE2T depletion resulted in a significant increase in the expression of the epithelial biomarker E-cadherin and simultaneous decreases in the expression of the mesenchymal biomarkers N-cadherin and vimentin. GRP78 over-expression reversed the effects of UBE2T depletion on EMT biomarker expression. GRP78 can affect the EMT signaling pathways related to Snail/Slug and TGF-β/Smad [32, 33]. These pathways are closely associated with invasion and migration of tumours [34]. EMT plays a key role in the acquisition of migratory activity by quiescent cells [35]. Tumour cells also can activate EMT biological processes, which increase stem cell properties and drug resistance and drive the metastasis and recurrence of cancer [36, 37]. The results revealed that UBE2T functions upstream of EMT. UBE2T depletion in GBM cells weakened their migratory and invasive abilities and ablated migration-related markers. These are key factors for GBM cell invasion and migration. GRP78 over-expression can reverse these effects. We concluded that UBE2T enhances GBM cell invasion and migration by modulating EMT via GRP78. Therefore, GBM recurrence and tumorigenicity might be regulated by the UBE2T/GRP78/EMT axis. This study also suggested the potential of the UBE2T/GRP78/EMT axis as a therapeutic target for GBM.

## MATERIALS AND METHODS

### Expression and significance of UBE2T in GBM

A total of 179 primary GBM samples (Supplementary Table 1B) and 125 recurrent GBM samples (Supplementary Table 1C) with RNA sequencing data and corresponding clinical information (Supplementary Table 1D) were obtained from the Chinese Glioma Genome Atlas (CGGA, http://www.cgga.org.cn). Another 1152 normal brain tissues (Supplementary Table 1E) with RNA sequencing data were obtained from the Genotype-Tissue Expression Project (GTEx). We merged the CGGA and GTEx datasets and identified differentially expressed genes (DEGs) using the normal brain tissue as control. Moreover, we researched 40 ubiquitination enzymes among the DEGs. To identify most meaningful gene, the GEPIA website was utilised to identify the survival data [38]. Multivariate analysis was conducted to evaluate the prognostic performance.

### Patient samples

In total, 9 normal brain tissues immunohistochemical sections, 43 GBM immunohistochemical sections, 9 frozen GBM tissues and 4 frozen normal brain tissues were obtained from Sanbo Brain Hospital, Capital Medical University (Beijing, China). All samples were gathered using protocols approved by the Ethics Committee of Sanbo Brain Hospital, and informed consent was obtained from all patients. The clinical and pathological classification and stage were ascertained using the WHO brain tumour criteria.

### Immunohistochemistry (IHC)

Tissue sections were deparaffinised, soaked in Tris-EDTA buffer (pH 9.0) boiled in a microwave and then incubated with antibodies against UBE2T (1:250; ab179802, Abcam), GRP78 (1:500; 11587-1-AP, Proteintech) and Ki-67 (1:500; sc-23900, Santa Cruz Biotechnology) at 4°C for 12 h. The next day, slides were washed, stained with secondary antibodies and 3, 3′-diaminobenzidine, counterstained with hematoxylin, dehydrated and mounted. The sections were reviewed and scored independently by two observers. The IHC score was determined on the basis of both the proportion of positively stained tumour tissue (%) and the intensity of staining (1, weak; 2, moderate; 3, strong) using the following formula: IHC score = percent of stained tumour × intensity. The cut-off value was 150 (low, <150; high, ≥150).

### Cell culture and plasmid and siRNA transfection

We used the human GBM cell lines U87, U118, LN229, U251 and U343 and the normal human astrocyte lines HEB and SVG p12. All cells were cultured in DMEM (Gibco, USA) supplemented with 10% foetal bovine serum (FBS) and maintained at 37°C in a fully humidified incubator with 5% CO_2_. Stable cell lines were generated via transfection of short hairpin RNA (shRNA) (shUBE2T-1, 5′- GATGCTTGATAATCTACCA-3′; shUBE2T-2, 5′-ATCCGATTTCTCACTCCAA-3′) (GenePharma) into LN229 and U251 cells using H1/GFP and Puro vectors. The cells were cultured for 14 days with 0.5 μg/mL puromycin after transfection. The over-expression plasmid FLAG-GRP78 was purchased from GeneCopoeia, and GRP78 siRNAs were obtained from GenePharma (siGRP78-1, 5′-GCCACCAAGAUGCUGACAUTT-3′; siGRP78-2, 5′-GGUUACCCAUGCAGUUGUUTT-3′). GRP78 siRNA and GRP78 plasmid transfection was performed using Lipofectamine 2000 (Invitrogen, USA) according to the manufacturer’s instructions.

### Transwell invasion and migration assays

We used Transwell insert chambers (Neuro Probe, USA) for these assays. In the migration assay, 3 × 10^4^ GBM cells were added to the upper chamber membrane (without Matrigel), which contained serum-free medium. The lower chambers were filled with medium containing 10% FBS. After 18–24 h, the migrated cells were fixed in methanol for 10 min, stained with crystal violet for 10 min, visualised via phase-contrast microscopy and photographed. Cells in six random microscopic fields (×100 magnification) were counted per well, and the mean was calculated. In the invasion assay, the upper chamber membrane containing 100 μL of 2% Matrigel (BD Biosciences, USA). The experiment was repeated three times.

### Immunoblotting (IB) and immunoprecipitation (IP)

For the immunoblotting assay, total cell protein extracts were prepared using PBS containing 1% NP-40 and a protease inhibitor cocktail (Roche). The lysates were separated using 10 or 12% SDS-PAGE and transferred to a polyvinylidene fluoride membrane. Actin and GAPDH antibodies were diluted 1:5000. The membrane was incubated with the indicated primary antibodies followed by anti-mouse or anti-rabbit secondary antibodies. Blotting membranes were re-probed with anti-actin antibody as a control. The antibodies used included anti-UBE2T (ab179802, Abcam), anti-GRP78 (11587-1-AP, Proteintech) and anti-GAPDH (60004-1-lg, Proteintech). In addition, anti-E-cadherin, anti-N-cadherin, anti-vimentin, anti-Ki-67, anti-actin and anti-mouse and anti-rabbit secondary antibodies were purchased from Cell Signaling Technology. The LAS-4000 luminescent image analyser (Fujifilm) was used to detect the chemiluminescence signal.

In the IP assay, cells were treated with IP buffer (20 mmol/L Tris/HCL, pH 7.6, 100 mmol/L NaCl, 20 mmol/L KCl, and 1.5 mmol/L MgCl_2_, 0.5% NP-40) containing a protease inhibitor cocktail. The total cell protein extracts were incubated with Protein A/G Sepharose beads (Roche) and pre-treated with anti-UBE2T (ab179802, Abcam) or anti-GRP78 (11587-1-AP, Proteintech) at 4°C overnight. The beads were washed with cell lysis buffer, and the immunoprecipitated samples were analysed via immunoblotting.

### Silver staining of SDS-PAGE blots and mass spectrometry analysis

SDS-PAGE analysis of UBE2T immunoprecipitates was performed as described previously. Moreover, the result of SDS-PAGE was checked via silver staining. Briefly, gels were fixed with 40% absolute alcohol/10% glacial acetic acid, washed with water and sensitised with 40% absolute alcohol containing 6.8 g of sodium acetate and 0.2 g sodium thiosulphate for 30 min. Gels were incubated without light in 0.1% (w/v) silver nitrate for 20 min and developed using 0.05% (w/v) paraformaldehyde in 3% (w/v) sodium carbonate until the desired staining had occurred. The reaction was stopped by the addition of EDTA sodium. Bands of interest in silver-stained gels were excised and analysed via mass spectrometry (CapitalBio Technology).

### Xenograft studies

Six-week-old BALB/c mice were purchased from Vital River Laboratories (Beijing, China). In total, 5 × 10^6^ LN229 cells transfected with shUBE2T-1 or control shRNA (ShVector) vectors were injected subcutaneously into mice. The tumour volume was measured every 5 days starting 2 weeks after transfection. After 1 month, the mice were sacrificed to measure the tumour volume, which calculated using the formula 1/2 × larger diameter × smaller diameter^2^. At the end of each experiment, tumours were paraffin-embedded, and 5.0-μm sections were excised and subjected to IHC staining. All animal care procedures and experiments were approved by the Institutional Animal Welfare Guidelines of Chinese Academy of Medical Sciences.

### Statistical analysis

In this study, R software (version 3.5.2) and GraphPad Prism 7.0 were used to conduct the analysis and generate graphs. Student’s *t*-test was used to assess the statistical significance of differences between experimental groups. The log-rank test and multivariate Cox regression analysis were used for the survival analysis. Pearson’s test was used to analyse the association of UBE2T expression with GRP78 expression. *P* < 0.05 was considered statistically significant.

### Availability of data and materials

All data generated or analyzed during this study are included in this published article and its supplementary information files.

## Supplementary Material

Supplementary Figure 1

Supplementary Table S1A

Supplementary Table S1B

Supplementary Table S1C

Supplementary Table S1D

Supplementary Table S1E
