# Supplementary material for: UBE2T promotes glioblastoma invasion and migration via stabilizing GRP78 and regulating EMT
Source: Aging (Albany NY). 2020 Jun 3;12(11):10275–89. doi: 10.18632/aging.103239 (PMC7346020; doi:10.18632/aging.103239)
Supplement: Supplementary Figure 1 [file aging-12-103239-s001.pdf]

SUPPLEMENTARY FIGURE

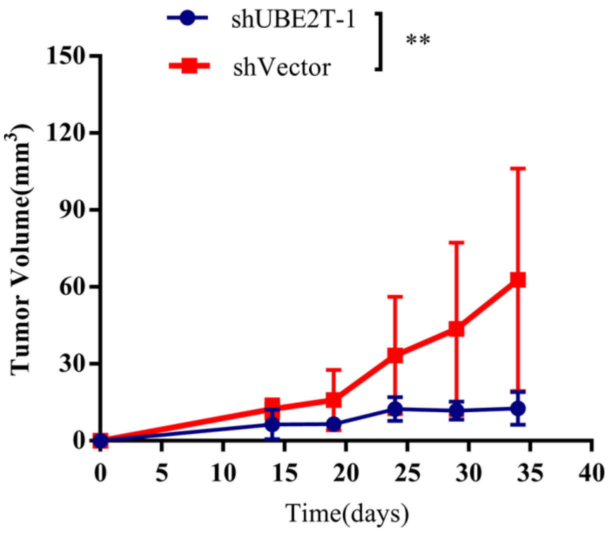

Supplementary Figure 1. UBE2T regulates the tumorigenesis of glioblastoma in nude mice. LN229 cells transfected with ShUBE2T-1 or the control vector (Sh Vector) growth curve of subcutaneous tumor volume in nude mice.
